# Pharmacological, behavioural and mechanistic analysis of HIV-1 gp120 induced painful neuropathy

**DOI:** 10.1016/j.pain.2007.02.015

**Published:** 2007-12-15

**Authors:** Victoria C.J. Wallace, Julie Blackbeard, Timothy Pheby, Andrew R. Segerdahl, Meirion Davies, Fauzia Hasnie, Susan Hall, Stephen B. McMahon, Andrew S.C. Rice

**Affiliations:** a Pain Research Group, Department of Anaesthetics, Pain Medicine and Intensive Care, Faculty of Medicine, Imperial College London, Chelsea and Westminster Hospital Campus, 369 Fulham Road, London SW10 9NH, UK; b Neurorestoration Group, Wolfson CARD, Kings College London, Guy’s Hospital Campus, London SE1 1UL, UK; c Department of Anatomy and Human Sciences, Kings College London, Guy’s Hospital Campus, London SE1 1UL, UK

**Keywords:** HIV-1, Neuropathy, Microglia, Macrophages, Gabapentin, Cannabinoids

## Abstract

A painful neuropathy is frequently observed in people living with human immunodeficiency virus type 1 (HIV-1). The HIV coat protein, glycoprotein 120 (gp120), implicated in the pathogenesis of neurological disorders associated with HIV, is capable of initiating neurotoxic cascades via an interaction with the CXCR4 and/or CCR5 chemokine receptors, which may underlie the pathogenesis of HIV-associated peripheral neuropathic pain. In order to elucidate the mechanisms underlying HIV-induced painful peripheral neuropathy, we have characterised pathological events in the peripheral and central nervous system following application of HIV-1 gp120 to the rat sciatic nerve. Perineural HIV-1 gp120 treatment induced a persistent mechanical hypersensitivity (44% decrease from baseline), but no alterations in sensitivity to thermal or cold stimuli, and thigmotactic (anxiety-like) behaviour in the open field. The mechanical hypersensitivity was sensitive to systemic treatment with gabapentin, morphine and the cannabinoid WIN 55,212-2, but not with amitriptyline. Immunohistochemical studies reveal: decreased intraepidermal nerve fibre density, macrophage infiltration into the peripheral nerve at the site of perineural HIV-1 gp120; changes in sensory neuron phenotype including expression of activating transcription factor 3 (ATF3) in 27% of cells, caspase-3 in 25% of cells, neuropeptide Y (NPY) in 12% of cells and galanin in 13% of cells and a spinal gliosis. These novel findings suggest that this model is not only useful for the elucidation of mechanisms underlying HIV-1-related peripheral neuropathy but may prove useful for preclinical assessment of drugs for the treatment of HIV-1 related peripheral neuropathic pain.

## 1 Introduction

Distal symmetrical polyneuropathy (DSP) afflicts 15–50% of people living with HIV-1 (Marshall et al., 2005; Verma et al., 2005), 50–60% of whom have measurable sensory abnormalities (Martin et al., 2003) and on-going, paroxysmal or stimulus evoked pain (Dalakas and Pezeshkpour, 1988; Martin et al., 1999). There are two predominant (and clinically similar) settings in which painful DSP may occur in HIV-disease. Firstly, a disease-related DSP associated with advanced disease and secondly a drug-induced DSP associated with the use of nucleoside reverse transcriptase inhibitors as part of an anti-retroviral therapy (Martin et al., 2003; Marshall et al., 2005). This study is solely concerned with the former scenario, which we will refer to as HIV-DSP. The pathophysiology of HIV-DSP is unclear: nerve biopsies and nerve conduction studies suggest axonal degeneration of both large and small diameter myelinated and unmyelinated axons and measurement of thermal thresholds reveals a high prevalence of thermal hypoesthsesia (and by implication a deficit in small fibre function) (Martin et al., 2003). Direct infection of the nervous system by HIV is thought to be unlikely (Michaels et al., 1988; Lipton, 1998) instead, HIV-1 appears to interact indirectly with neurons via binding of the external envelope protein, gp120, to the chemokine receptors CXCR4 and/or CCR5. In line with this, HIV-1 gp120 causes apoptosis in various central nervous system (CNS) neuronal populations *in vitro* (Lipton, 1991; Bennett et al., 1995) and *in vivo* (Bagetta et al., 1996; Corasaniti et al., 2001) and results in marked pain-like behaviour when injected into the spinal intrathecal space in rats (Milligan et al., 2000) or mice (Minami et al., 2003). In the peripheral nervous system, HIV-1 gp120 can influence the activity (Oh et al., 2001) and survival (Keswani et al., 2003) of sensory neurons and has recently been shown to interact directly with axons *in vitro* leading to toxicity (Melli et al., 2006). Accordingly, peripheral administration of gp120 is associated with the development of pain-like behaviour in rats (Herzberg and Sagen, 2001) suggesting the possibility that HIV-1 gp120 interactions with the peripheral nerve may be a causative factor in the generation of peripheral neuropathic pain in humans. In an effort to elucidate HIV-associated neuropathic pain pathophysiology we have pharmacologically and mechanistically characterised the effects of perineural delivery of HIV-1 gp120 in rats and associated pain and co-morbidity behaviour. To draw parallels between this model and data from human studies, we have pharmacologically validated the associated behavioural sensitivity with drugs known to possess analgesic efficacy in other rodent models of neuropathic pain and from randomised controlled trials conducted in humans, including amitriptyline, gabapentin, morphine and the cannabinoid agonist WIN-55,212-2. Finally, we have assessed the effects of perineural HIV-1 gp120 on: peripheral nerve morphology, innervation of sensory target tissues, sensory neuronal phenotype, and the activity of non-neuronal, particularly immune, cells in the PNS and CNS.

## 2 Materials and methods

### 2.1 Animals and surgical methods

All experiments conformed to the British Home Office Regulations and IASP guidelines (Zimmermann, 1983). Male Wistar rats weighing 200–250 g were used for all experiments (B & K, Hull, UK) and were housed in a temperature-controlled environment, maintained on a 14:10 h light–dark cycle (experiments were performed during the light phase) and provided with feed and water *ad libitum*. Under 1–2% isoflurane anaesthesia (Abbott, UK) in 1% O_2_ and 1% N_2_O, and aseptic surgical conditions, the left sciatic nerve was exposed in the popliteal fossa without damaging the perineurium. In six rats a partial sciatic nerve injury (PSNI) (Seltzer et al., 1990) was performed in which 1/3–1/2 of the sciatic nerve was tightly ligated using 7.0 suture thread (Ethicon, Edinburgh, UK). The wound was then closed with 4.0 sutures and animals allowed to recover. For perineural HIV-1 gp120 delivery, the nerve was initially exposed to the HIV-1 gp120 protein or vehicle control by placing a 5 mm × 2 mm piece of gel foam (Ethicon) soaked in saline containing 0.1% rat serum albumin (RSA) (Sigma, Poole, UK) and 200 ng HIV-1 gp120-MN (>95% pure; Immunodiagnostics, Bedford, MA) or saline containing just RSA in direct contact with the nerve to form a pool of protein solution around the nerve and left in place for 30 min. Following this, oxidised regenerated cellulose (Surgicell, Ethicon), previously soaked until saturation in the same 200 μl of saline containing 200 ng gp120-MN or RSA, was wrapped loosely around the sciatic nerve 2–3 mm proximal to the trifurcation so as not to cause any nerve constriction and left *in situ* (modified from (Herzberg and Sagen, 2001)). The nerve was gently manipulated back into place and the muscle and skin incisions closed with 4/0 silk sutures (Ethicon). In both surgical models 0.05 ml of 0.25% Bupivacaine (STR, UK) was administered to the wound s.c. and 5 mg/kg carprofen administered i.p. 3–4 h post-operatively.

### 2.2 Behavioural reflex testing

The threshold for hind paw withdrawal in response to graded mechanical stimulation was measured in conscious animals using two types of device. Firstly, graded von Frey nylon filaments (Alan Ainsworth, UCL, London), which were used to deliver a calibrated indentation pressure against the hairless skin of the hind paws and for which the threshold response was defined by the nominal bending force of the filament that evoked foot withdrawal at least three times in every five applications when delivered at a rate of 1 Hz (Chaplan et al., 1994). Secondly, an electronic von Frey device (Ahmad and Rice, 1999) of 0.5 mm^2^ probe tip area (Somedic Sales AB, Sweden) applied manually at a rate of 8–15 g/s to the mid-plantar surface of the hind paw with the withdrawal threshold (g) defined as the average force that evoked an active limb withdrawal response over five applications. As the electronic von Frey device is less well characterised, we chose to use calibrated von Frey hairs as a validation of its sensitivity to detect threshold changes. The time for hind paw withdrawal in response to a quantified noxious heat stimulus was assessed using the Plantar test (Ugo Basile, Italy) (Hargreaves et al., 1988). The thermal stimulus (set at an infrared intensity that produced standard latency of approx. 10 s) was applied to the mid-plantar surface of the hind paw and the latency (seconds) to withdrawal recorded over three applications. The presence of a behavioural correlate of cold allodynia was assessed using the acetone drop application technique (Bridges et al., 2001). A single drop of acetone was applied via a 1 ml syringe to the mid-plantar surface of each hind paw and the outcome defined as the percentage of applications which evoked an active limb withdrawal from a total of five applications.

Baseline measurements were obtained for all animals (*n* = 12 per group) over the course of a week prior to surgery. Behavioural reflex tests were then carried out in a blinded and randomised manner following surgery to identify the development of any reflex sensitivity in treated animals. The threshold value at each time point tested was calculated as the mean ± SEM.

### 2.3 Open field activity

At days 7 and 14 post surgery, rats (*n* = 8 per group) were placed into a 1 m × 1 m arena illuminated to 4 lux with a defined inner zone of 40 cm × 40 cm. Locomotion of the rats within the arena was tracked over a 15 min period recorded using a Sanyo VCB 3372 high-resolution monochrome camera (Tracksys, Notts, UK) and stored and analysed with Ethovision software v.3 (Tracksys). The total distance moved, time spent in the inner zone and the number of entries into the inner zone were calculated and displayed as the mean ± SEM. For HIV-1 gp120 treated animals, only those with mechanical hypersensitivity (a change of at least 30% from baseline) as demonstrated using the electronic von Frey device were included (100% inclusion rate).

### 2.4 Pharmacological manipulation

For all experiments, animals were block randomised to treatment groups and the experimenter blinded to drug treatments received. Behavioural responses to punctuate mechanical stimuli were measured as described previously using an electronic von Frey device. Baseline measurements were obtained at least twice prior to surgery and post surgical values determined at days 11 and 14 post surgery. Only animals that developed mechanical hypersensitivity of at least 30% change were used for pharmacological experiments (drop out rate of 10% with resulting *n* = 8 per group). To test the effect of chronic treatment with known analgesics, we chose doses known to be effective in rats limiting the need for dose–response studies. Animals were administered the drug or vehicle twice daily (8am and 6pm) on post surgery days 15–18, and behavioural tests performed once each day 1–2 h following the first injection. The following drugs were tested: morphine sulphate (Sigma; 2.5 mg/kg in 0.5 ml saline i.p. b.d.); gabapentin (Pfizer Ltd 30 mg/kg in 0.5 ml saline i.p. b.d.); the mixed CB_1_/CB_2_ cannabinoid receptor agonist WIN 55,212-2 [(mesylate (R)-(+)-(2,3-dihydro-5-methyl-3-(4-morpholinylmethyl)pyrrolo(1,2,3-de)-1, 4-benzoxazin-6-yl)-1-naphthalenylmethanone) (Sigma) 2.5 mg/kg in 0.75 ml saline with 40% dimethylsulphoxide (DMSO) i.p. b.d.]; and amitriptyline (Sigma 10 mg/kg in 0.5 ml saline i.p. b.d.) (Hasnie et al., 2007).

### 2.5 Morphological investigations

Nerve morphology was examined in animals after sciatic nerve exposure to HIV-1 gp120 (*n* = 8) or RSA (*n* = 6). At 7 (*n* = 7) and 14 (*n* = 7) days post surgery, 4 cm lengths of sciatic nerve were removed from the experimental site. Under a dissecting microscope, a length of nerve that included the wrap site, as determined by the localised discolouration of nerve associated with resorbing oxidised cellulose, was removed, attached to a card in order prevent shrinkage during processing, and immersion fixed in 3% glutaraldehyde in 0.1 M phosphate buffer, pH 7.4. Specimens were subsequently washed, postfixed in phosphate buffered OsO_4_ and processed through to embedding in TAAB medium embedding resin (TAAB Laboratories Equipment Ltd, Aldermaston, UK) for transverse sectioning. Each nerve was divided in order to examine the centre of the site of gp120 or RSA application, and sites within 1 cm proximal and distal to this point. Transverse 1 μm sections were subsequently stained with toluidine blue and assessed under oil immersion using a Zeiss Axioplan2 light microscope. The assessor was blinded to the treatment group for analysis. From the same blocks assessed at LM, ultrathin sections were saturated with uranyl acetate and lead citrate, and viewed on the Hitachi H7600 electron microscope. For each experimental group, six scattered fields of nerve were chosen at random and photographed for the assessment of morphology by a blinded observer.

### 2.6 Immunohistochemistry

Rats were deeply anaesthetised with pentobarbitone and transcardially perfused with heparinised saline followed by 4% paraformaldehyde (Sigma) in 0.1 M PB (Sigma). The lumbar spinal cord, the treated sciatic nerve, the L4 and L5 DRGs and the appropriate glabrous skin, excised bilaterally from the wide part of the plantar hind paws (i.e., within the dermatomes for sciatic nerve innervation), were removed. All tissue was postfixed with the same fixative for 4 h and transferred to 15% sucrose in 0.1 M PB for 2 h and left overnight in 30% sucrose in 0.1 M PB. It was then embedded in optimum cryostat temperature (OCT) mounting medium (VWR, Poole, UK) and frozen over liquid nitrogen. Cryostat sections of DRG (12 μm), L5 spinal cord (transverse, 20 μm), sciatic nerve (longitudinal, 15 μm) and hind paw skin (transverse, 15 μm) were thaw-mounted on glass slides pre-coated with poly-l-lysine (VWR).

For all fluorescence immunohistochemistry, sections were pre-incubated in buffer (0.1 M phosphate buffered saline (PBS), pH 7.4, containing 0.2% Triton X-100) containing 10% normal donkey serum for 1 h at room temperature and incubated with primary antibodies diluted in buffer containing 4% donkey serum overnight.

Antisera were used at the following concentrations; rabbit anti-ATF3 (1:400; Santa Cruz Biotechnology, Santa Cruz, CA), mouse anti p-c-Jun (1:500; Santa Cruz), rabbit anti-calcitonin gene related peptide (CGRP) (1:4000; Chemicon, Hampshire, UK); rabbit anti-NPY (1:1000, Peninsula Laboratories Inc, Belmont, CA); rabbit anti-galanin (1:2000; Peninsula, Cambridgeshire, UK); rabbit anti-growth associated protein-43 (GAP-43) (1:1000; Chemicon,); rabbit anti-cleaved caspase-3 (1:250; New England Biolabs, Hitchin, UK); rabbit anti-transient receptor potential vanilloid 1 (TRPV1) (1:5000; Abcam Plc, Cambridge, UK) mouse monoclonal anti-neurofilament 200 kDa (NF200) (1:2000; clone N52; Sigma); mouse anti-rat CD68 (ED1) (1:2500; Serotec, Oxford, UK); rabbit anti-glial fibrillary acidic protein (GFAP) (1:1000; Chemicon); rabbit anti-PGP 9.5 (1:1000; Ultraclone, Isle of White, UK); mouse anti-rat OX-42 (1:800; Serotec). Sections were then washed in buffer and incubated with the appropriate secondary antibodies linked to either isothiocyanate (Cy3) (donkey anti-mouse-Cy3 1:300; Jackson ImmunoResearch Laboratories, Westgrove, PA) or fluorescein isothiocyanate (FITC) (donkey anti-rabbit-FITC 1:300; Jackson ImmunoResearch) for 2 h at room temperature. Three final washes in 0.1 M PBS were conducted before cover-slipping with Vecta-Shield (Vector Laboratories, Peterborough, UK) for analysis. Control sections were processed as above omitting the primary antisera.

For biotinylated labelling of microglia, sections were pre-incubated with 0.3% H_2_0_2_ for 15 min, washed in PBS and incubated with mouse anti-OX42 overnight. Sections were then washed in PBS and incubated with biotinylated horse anti-mouse antibody (1:400; Jackson ImmunoResearch) for 90 min. After a further wash in PBS, sections were incubated with the avidin–biotin complex (Vector Labs) for 2 h, washed as above, and the reaction developed with 3,3,-diaminobenzidine (DAB; Vector Labs). Sections were then dehydrated in increasing concentrations of alcohol (50–100%), cleared with xylene and cover-slipped with DePex mounting medium (VWR).

### 2.7 Image analysis and quantification

All analysis was performed by an observer who was unaware of the experimental group of the tissue being examined. Fluorescent images were visualised using a Leica (Leica, Milton Keynes, UK) fluorescence microscope, equipped with the appropriate filter blocks and captured at consistent exposure times using a Hamamatsu CCD camera and analysed using Leica QWin v.3.0 software (Leica). For DRG, analysis was performed at 20× or 40× magnification on 6–8 randomly selected 12 μm sections of DRG (separation of 180 μm) from each of four to six animals per group (ipsilateral to HIV-1 gp120, contralateral to HIV-1 gp120 and ipsilateral to RSA), only neurons with clear nuclei were counted. In each case, the analysis was performed in an automated manner using constant detection threshold levels determined from positive control tissue (the DRG of rats 7 days following PSNL surgery). The total number of neurons per section was determined by counting both NF-200 labelled and non-labelled neuronal cell bodies and number of DRG neurons expressing immunoreactivity (IR) for ATF3, p-c-Jun, CGRP, NPY, galanin TRPV1, caspase-3 or GAP-43 reported as a percentage of total number of neurons/section. In some cases, the proportion of co-localised cells was also calculated. To quantify alterations in satellite cell morphology within the DRG, the, number of neuronal cell bodies associated with GFAP-IR was counted and expressed as a percentage of total DRG neurons per section. To quantify the activated or infiltrating macrophages in DRG, regions of the DRG containing only sensory neuronal cell bodies (excluding peripheral nerve) were analysed in a defined area (450 μm × 450 μm) within which the number of CD68-IR cellular profiles was counted and expressed as the total number of CD68-IR cellular profiles per unit area. For the assessment of CD68-IR in the sciatic nerve, six sections from each of six animals per group were captured in three places; at the site of HIV-1 gp120 or RSA application, 1 cm distal to or 1 cm proximal to application and analysis performed in an area encompassing only nerve and not perineurium or remnants of oxidised cellulose. To quantify CD68-IR in the sciatic nerve, the number of CD68-IR cellular profiles was measured and expressed as total number of CD68-IR cellular profiles per unit area (450 μm × 250 μm). To quantify epidermal nerve fibre linear density (McArthur et al., 1998), PGP 9.5-immunoreactive nerves were counted in the epidermis of four footpad sections from each of four animals per group at a magnification of 20× using previously described methods (Hsieh et al., 2000; Lin et al., 2001; Keswani et al., 2006). Each individual nerve with branching points inside the epidermis was counted as one. For epidermal nerves with branching points in the dermis, each individual nerve was counted separately. The total length of the epidermis along the upper margin of the stratum corneum in each footpad was measured and epidermal nerve density was expressed as the number of fibres/mm of epidermal length.

Analysis of spinal cord immunoreactivity was performed on a minimum of five randomly selected L5 coronal spinal cord sections from each of four animals per group (HIV-1 gp120 treated, RSA sham treated or naïve) at 20× magnification. The percentage area of fluorescent-labelled ipsilateral and contralateral GFAP or OX-42 staining in the dorsal horn (laminae I–IV) was determined in a defined area of 860 μm × 580 μm using an automated grey-scale detection system and expressed as means % ± SEM (Blackbeard et al., 2005). Analysis of the ipsilateral and contralateral dorsal horns of biotin-labelled spinal cord sections was performed at 20× magnification and the activation state of the microglia assessed according to a modified rating scale and guideline (Colburn et al., 1997), where 0 represents the resting state, and 3 represents maximal activation and displayed as the mean score (±SEM) over five sections from each of four animals per group.

### 2.8 Statistical analysis

Sigmastat version 2.03 (SPSS Inc, Surrey, UK) was used to determine the presence of statistically significant differences (*P* < 0.05) throughout the study. For behavioural reflex measures and open field activity, all groups were compared using a one-way analysis of variance (ANOVA) with Dunn’s all pairwise multiple comparisons *post hoc* analysis. For immunohistochemical and pharmacological studies, experimental groups were compared using a Kruskal–Wallis one-way ANOVA on Ranks with an all pairwise multiple comparisons procedure (Dunn’s Method). In all cases, the investigator was blinded to the experimental status of each animal.

## 3 Results

### 3.1 Perineural HIV-1 gp120 causes mechanical but not thermal or cold hypersensitivity

**Fig. 1:**
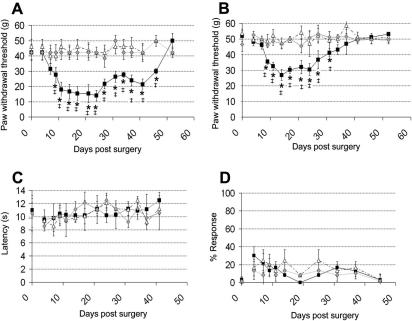
The development of reflex sensitivity to punctate mechanical stimuli, but not to thermal or cold stimuli, in the limb ipsilateral to perineural HIV-1 gp120 treatment of the sciatic nerve. (A–D) Hind paw withdrawal thresholds to (A) graded von Frey filaments, (B) an electronic von Frey device, (C) a noxious thermal stimulus and (D) an acetone drop, measured following perineural HIV-1 gp120 treatment of the sciatic nerve (*n* = 12) or perineural RSA (sham) treatment of the sciatic nerve (*n* = 12). Statistical significance of differences between paws ipsilateral (■) and contralateral () to HIV-1 gp120 and ipsilateral to RSA (▵) (^∗^*P* < 0.05) was determined by a one-way ANOVA with Dunn’s all pairwise multiple comparisons or (^‡^*P* < 0.05) between each value and the relevant baseline value (at time 0) using a one-way ANOVA with Dunnett’s multiple comparisons versus control post hoc analysis. Each value is the mean ± SEM.

Similar to other well-characterised models of neuropathic pain, rats exposed to perineural HIV-1 gp120 developed a long lasting, but reversible, mechanical hypersensitivity of the ipsilateral hind paw as measured by (a) calibrated graded von Frey filaments (gvF) (Fig. 1A), and (b) the electronic von Frey device (evF) (Fig. 1B) as compared to the contralateral hind paw or RSA (sham) treated rats. The heightened sensitivity to both tests was significant from day 9 after HIV-1 gp120 treatment, but reached peak values from day 12 until day 21 post-surgery [average threshold between days 12 and 21: gvF = 15.3 ± 0.4 g (36% of BL) and evF = 30.4 ± 1.0 g (56% of BL)]. The hypersensitivity reversed to baseline values by day 52 post surgery. No such hypersensitivity was apparent in the contralateral hind paw or in sham animals. These tests also confirmed that the electronic von Frey device is a reliable and sensitive test for the detection of mechanical threshold changes and therefore, we used only this apparatus for the remainder of mechanical reflex testing. In contrast to most nerve injury induced models of neuropathic pain, there was no evidence for the development of sensitivity to either thermal (Fig. 1C) or cold stimuli (Fig. 1D). We observed no overt motor deficit following HIV-1 gp120 treatment which was further confirmed by measuring spontaneous locomotor activity in the open field area (see below).

### 3.2 HIV-1 gp120 treatment results in altered spontaneous locomotor activity in the open field arena

**Fig. 2:**
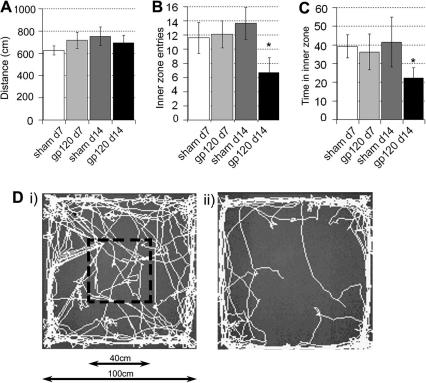
Spontaneous exploratory activity as measured in an open field arena is altered in rats 14 days following perineural HIV-1 gp120 treatment of the sciatic nerve. (A) The total distance moved within the open field arena (1 m × 1 m) was assessed over 15 min and is not significantly altered in animals 7 and 14 days following perineural HIV-1 gp120 treatment as compared to perineural RSA (sham) treatment (*n* = 8 per group). (B) The number of entries into the inner zone (40 × 40 cm) and (C) time spent in the inner zone of the open field arena were significantly reduced in rats 14 days following perineural HIV-1 gp120 treatment as compared to following perineural RSA (sham) treatment and to both treatment groups 7 days post surgery. The statistical significance of differences between all groups (^∗^*P* < 0.05) was determined using a one-way ANOVA with Dunn’s all pairwise multiple comparisons. Each value is the mean ± SEM. (D) Example traces of (i) sham versus (ii) HIV-1 gp120 treated rats over 15 min in the open field arena.

As a novel measure of pain related behaviour that does not rely on reflex thresholds, we assessed the spontaneous exploratory activity of our rats in the open field activity paradigm which has been extensively employed for the assessment of anxiolytic agents in rodents (Carli et al., 1989; Holmes, 2001; Cryan and Holmes, 2005). As an indicator of normal locomotion, there was no significant difference in the total distance moved by HIV-1 gp120 treated animals at day 7 or 14 post surgery versus sham animals (Fig. 2A), suggesting a lack of overt motor deficit. However, at day 14 post surgery exploration of the centre of the arena (as calculated by the number of entries into the inner zone and the time spent in the inner zone) was significantly decreased in HIV-1 gp120 treated animals as compared to shams or to either treatment group at day 7(Fig. 2B–D). This lack of exploration was associated with thigmotactic (‘wall-hugging’ behaviour) (Fig. 2D).

### 3.3 HIV-1 gp120 associated mechanical hypersensitivity is selectively responsive to clinically effective analgesic drugs

**Fig. 3:**
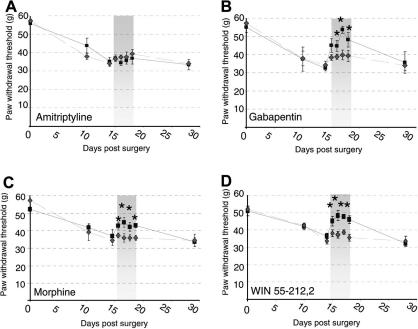
Effects of systemic administration of analgesic compounds on sensitised behavioural reflex responses following perineural HIV-1 gp120 treatment. Paw withdrawal thresholds in response to cutaneous punctate mechanical stimulation with an electronic von Frey device were measured in animals following perineural HIV-1 gp120 to one sciatic nerve and the effect of drugs delivered at the time of peak behavioural change assessed. Ipsilateral values are displayed before, during and after i.p. administration of each pharmacological agent (■) versus the appropriate vehicle () as paw withdrawal threshold (g). Drug administration period (days 15-18) represented by shaded area. For all measurements, each value is the mean ± SEM and statistical significance (^∗^*P* < 0.05) of any differences between drug and vehicle threshold values was determined by a Kruskal–Wallis one-way ANOVA with Dunn’s all pairwise multiple comparisons group post hoc analyses. (A) Amitriptyline (10 mg/kg i.p. b.d.) (*n* = 8) had no significant effect on behaviour versus the saline vehicle control (*n* = 8). (B) Gabapentin (30 mg/kg i.p. b.d.) (*n* = 8); (C) Morphine (2.5 mg/kg i.p. b.d.) (*n* = 8); and (D) the mixed CB1/CB2 cannabinoid receptor agonist WIN 55,212-2 (2 mg/kg i.p. b.d.) (*n* = 8) all significantly reversed behavioural reflex sensitivity to mechanical stimulation versus the appropriate vehicle control (*n* = 8).

To assess the sensitivity of the perineural HIV-1 gp120 induced mechanical hypersensitivity to clinically efficacious analgesic compounds, we have evaluated the analgesic efficacy of amitriptyline, morphine, gabapentin and the cannabinoid receptor agonist, WIN-55,212-2, in our model. When administered between days 15 and 18 post perineural HIV-1 gp120 administration, amitriptyline (10 mg/kg i.p. b.d.) was not associated with any significant reversal of the heightened sensitivity to punctuate mechanical stimuli as compared to vehicle administration (Fig. 3A). In contrast, gabapentin (30 mg/kg, i.p. b.d. on days 15–18) significantly reversed the mechanical hypersensitivity observed at day 14 (Fig. 3B) (d14 32.8 ± 1.9 g; d18 = 48.1 ± 1.7 g). Vehicle administration had no significant effect. Similarly, morphine (2.5 mg/kg i.p b.d. on days 15–18) significantly reversed the mechanical hypersensitivity displayed at day 14 (Fig. 3C) (d14 37.0 ± 3.6 g; d18 = 43.1 ± 1.3 g). Finally, systemic administration of the mixed CB_1_/CB_2_ cannabinoid receptor agonist WIN 55,212-2 (2 mg/kg, i.p. b.d.) significantly reversed the mechanical hypersensitivity displayed at day 14 (Fig. 3D) (d14 36.5 ± 1.6 g; d18 = 46.1 ± 2.3 g). The vehicle (40% DMSO) had a small but significant effect on behaviour, however, the analgesic effect of WIN 55,212-2 was significantly greater indicating an effect of the drug above the vehicle. For all drugs tested, mechanical hypersensitivity was re-established by day 28, indicating reversal of the drug effect and re-establishment of the neuropathic state.

### 3.4 LM and EM analysis reveals that overt axonal damage is not a consequence of perineural HIV-1 gp120 treatment

**Fig. 4:**
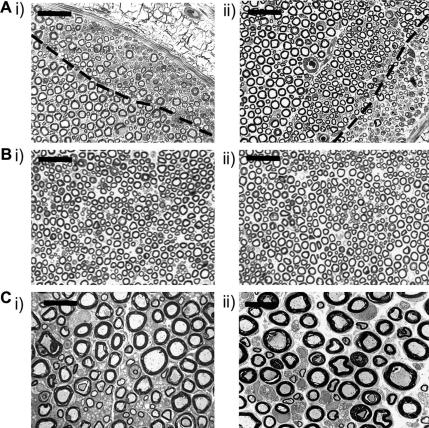
Effects of perineural treatment with RSA or HIV-1 gp120 on the integrity of axons in the sciatic nerve. (A–B) Toluidine blue-stained resin sections (1 μm) from the site of application via an oxycellulose wrap of (i) RSA and (ii) HIV-1 gp120 to the sciatic nerve on day 14 post treatment. (A) Examples of the sciatic nerve highlighting small areas of damage which occurred in the perineural window of the nerve. The broken line demarcates the area of morphologically normal nerve from the damaged area. Scale bar = 20 μm. (B) Examples of nerve in which axons appear undisturbed. (C) Examples of ultrathin sections of nerve within 1cm distal to the site of application of (i) RSA and (ii) HIV-1 gp120 to the sciatic nerve on day 14 post treatment assessed by EM representing the majority of the nerve where myelinated and unmyelinated axons appear normal. Scale bar = 10 μm.

To determine the extent of any damage to myelinated axons in response to either the enwrapment protocol or specifically in response to perineural HIV-1 gp120 treatment, nerves were examined by light microscopy prior to (day 7) and at the time of peak behavioural change (day 14) in sham and treated animals. There was evidence of either primary demyelination or Wallerian degeneration at 7 days, and of remyelination of affected axons (as assessed by visual examination) at 14 days, in both RSA (*n* = 6) and HIV-1 gp120 (*n* = 8) treated nerves at the site of application of the oxidised cellulose wrap. Affected axons tended to lie immediately deep to the perineurium (Fig. 4A) in a pattern similar to that seen in the “perineurial window” experimental model (Sugimoto et al., 2002): this suggests that at least some of the changes were attributable to the enwrapment protocol and that they occurred secondary to a disturbance of perineurial integrity and/or of the microvascular bed of the nerve. Damage was often confined to the axons in small outlying fascicles that had branched proximally from the parent sciatic nerve: these fascicles commonly displayed clear evidence of discontinuities of the perineurial cellular layers that were consistent with a perineurial window effect. Scattered degenerating axons were occasionally seen lying deeper within the endoneurium in sham and treated animals; however the majority of axons appeared normal (Fig. 4B). In marked contrast, the large nerve fascicles appeared normal. Inspection at the EM level of the day 14 tissue from sites just distal to, and at, the application site confirmed the LM findings in both endoneurium and perineurium. Bundles of regenerating unmyelinated axons were seen within areas of the endoneurium that contained remyelinating axons, but there was no evidence of degeneration of unmyelinated axons within endoneurium that appeared to be “morphologically normal” (Fig. 4C). Importantly, there was no apparent difference to the extent and frequency of signs of axonal damage between sham and HIV-1 gp120 treated animals implying that this minimal damage is associated with the surgical preparation.

### 3.5 Perineural HIV-1 gp120 is associated with decreased intraepidermal nerve fibre density

**Fig. 5:**
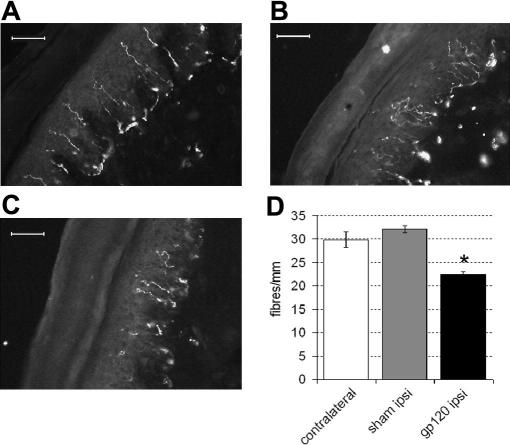
Intraepidermal nerve fibre density is decreased following perineural HIV-1 gp120. (A–C) Examples of PGP-9.5-immunoreactive profiles (A) contralateral to HIV-1 gp120, (B) ipsilateral to RSA or (C) ipsilateral to HIV-1 gp120 at day 14. (D) Immunohistochemical analysis of the glabrous skin of the hind paw reveals decreased IENF density ipsilateral to perineural HIV-1 gp120 by day 14 (*n* = 4) as compared to hind paw contralateral to treatment (*n* = 4) or ipsilateral to sham teatment (*n* = 4). Statistical significance of differences between groups (*P < 0.05) was determined by an ANOVA with Dunn’s all pairwise multiple comparisons. Each value is the mean ± SEM. Scale bar = 30 μm.

Immunostaining of skin biopsies for the neuronal marker protein gene product 9.5 (PGP 9.5), a ubiquitin carboxyl hydrolase (McCarthy et al., 1995; Kennedy et al., 1996), can be used reliably and reproducibly to quantify the intraepidermal nerve fibre (IENF) density (Smith et al., 2005) in humans and rodents (McCarthy et al., 1995; Herrmann et al., 1999; Lin et al., 2001; Keswani et al., 2006). Intraepidermal nerve fibres, which represent the terminals of C (nociceptive) fibres, are known to degenerate in patients with HIV-SN (Polydefkis et al., 2002). We therefore employed such immunohistochemical methods to quantify IENF densities in the lateral plantar surface of the hind paw of rats (i.e., the area innervated by the sciatic nerve (Decosterd and Woolf, 2000)) treated with perineural HIV-1 gp120. At 14 days following perineural HIV-1 gp120, we found there to be significantly fewer nerve fibres ipsilateral to injury (22.5 ± 0.5/mm) as compared to sham treated (32.1 ± 0.8/mm) or contralateral foot skin (29.7 ± 1.6/mm) (Fig. 5).

### 3.6 Perineural HIV-1 gp120 is associated with increased numbers of macrophages in the nerve

**Fig. 6:**
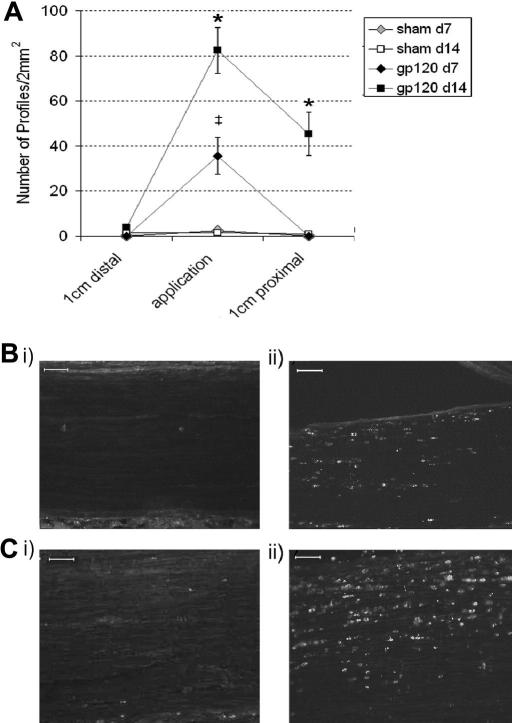
The presence of macrophages in the HIV-1 gp120 treated sciatic nerve. (A) Immunohistochemical analysis of the sciatic nerve reveals the presence of immunoreactivity for the macrophage marker CD68 at the site of administration of HIV-1 gp120 by day 7 (♦*n* = 6) and day 14 (■ *n* = 6) as well as a spread of macrophages into the distal nerve by day 14 as compared to no significant CD68-IR in the RSA (sham) treated nerve at day 7 (*n* = 6) or day 14 (□ *n* = 6). Statistical significance of differences between groups (*P* < 0.05) was determined by an ANOVA with Dunn’s all pairwise multiple comparisons where * represents significant difference from all other group and ‡ represents significance from the appropriate sham control group. Each value is the mean ± SEM. (B–C) Examples of CD68-immunoreactive profiles at the site of (i) RSA or (ii) HIV-1 gp120 administration at (B) day 7 and (C) at day 14. Scale bar = 100 μm.

Macrophage infiltration and activation are cardinal features of Wallerian degeneration in the PNS (Myers et al., 1996; Abbadie et al., 2003). At days 3, 7 and 14 post application, we assessed the presence of macrophages in the HIV-1 gp120 treated sciatic nerve at the site of application as well as 1 cm proximally and 1 cm distally, using an antibody to CD68 (ED-1), a lysosomal protein expressed by activated tissue macrophages. By day 3, there was no evidence of activated macrophages within the nerve. By day 7 there was a small but significant increase in the number of macrophages present in and around the site of HIV-1 gp120 application as compared to sham treated sciatic nerve (35.2 versus 2.5 profiles per 2 mm^2^) (Fig. 6A and B). At this time point, no macrophages were detectable proximal or distal to the application site or in the contralateral nerve. By day 14 the number of macrophages in the HIV-1 gp120 treated nerve had increased by more than 100% at the site of application, whereas sham levels remained low (Fig. 6A and C). Furthermore, at day 14, there was a significant spread of CD-68-IR macrophages into the proximal region of the HIV-1 gp120 treated nerve which was not apparent in the sham or in the distal portion of the nerve in any treatment group (Fig. 6A). By day 45 post HIV-1 gp120 application, CD68-IR had returned to basal levels (data not shown).

### 3.7 Perineural HIV-1 gp120 is associated with increased ATF3, caspase-3 but not GAP-43 or c-Jun expression in the DRG

**Fig. 7:**
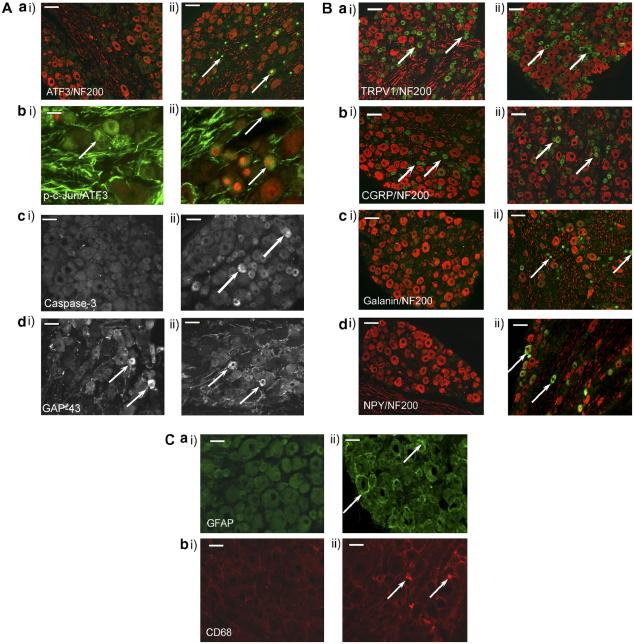
Immunohistochemical characterisation of the L5 DRG 14 days following perineural HIV-1 gp120. (A) Examples of the neuronal expression of (a) ATF3 (green) co-stained for NF-200 (red); scale bar = 100 μm and (b) p-c-Jun (green) co-stained for ATF3 (red); scale bar = 30 μm (c) caspase-3 and (d) GAP-43 with particular reference to high expressing cells; scale bar = 50 μm. (B) Examples of the neuronal expression of (a) TRPV1 (green), (b) CGRP (green), (c) galanin (green) and (d) NPY (green), all co-stained for NF-200 (red) with co-localisation appearing in yellow; scale bar = 100 μm. (C) Examples of the non-neuronal cell expression of (a) the satellite cell marker GFAP (green) and (b) the macrophage marker CD68 (red); scale bar = 50 μm. Arrows indicate examples of cells immunoreactive for the protein of interest. All images are from the L4/L5 dorsal root ganglia of animals exposed to (i) perineural RSA at day 14 versus (ii) perineural HIV-1 gp120. There was a statistically significant increase in the number of cells expressing ATF3, caspase-3, galanin, NPY, GFAP and CD68 in the DRG ipsilateral to perineural HIV-1 gp120 (*n* = 4–6) as compared to DRG ipsilateral to perineural RSA (*n* = 4–6). No change was observed in p-c-Jun, GAP-43, TRPV1 or CGRP expression.

By day 7, a small but significant proportion of cells (8.6%) in the DRG ipsilateral to perineural HIV-1 gp120 were immunoreactive for ATF3 as compared to the contralateral DRG and DRG ipsilateral to perineural RSA (Table 1). The majority of ATF3-IR neurons were not co-localised with NF-200 indicating that they were small diameter C-fibre neurons. By day 14, 26.9% of DRG neurons ipsilateral to perineural HIV-1 gp120 were ATF3-IR compared to 4.6% ipsilateral to perineural RSA and 0.8% in the contralateral DRG (Table 1). Furthermore, co-localisation with NF-200 indicated that 85% of ATF3 expressing neurons ipsilateral to HIV-1 gp120 at day 14 were small diameter C-fibre neurons (1252 of 1483 ATF3-IR cells were NF-200 negative) (Fig. 7A, a).

The increased expression of ATF3 at day 14 led us to assess the DRG at day 14 for markers of apoptosis. We investigated the presence of the pro-apoptotic proteins; c-Jun and caspase-3 in the sensory neurons in the DRG at times of peak behavioural sensitivity. Using a phospho-specific antibody to detect the activated (phosphorylated) form of c-Jun (p-c-Jun) (Seijffers et al., 2006) we found there to be no significant upregulation of the protein in DRG from HIV-1 gp120 treated animals as compared to sham animals (Fig. 7A, b and Table 2). The small number of cells expressing this protein in both experimental groups is in line with previous data indicating that activated c-Jun is present in some naïve non-injured neurons (Seijffers et al., 2006). However, there was a significant expression of caspase-3 in 25.3% of cells ipsilateral to treatment as compared to no significant expression in contralateral DRG or in sham DRG (Fig. 7A, c and Table 2). Similar to ATF3 expression in sections from the same DRG, the majority of caspase-3 cells were small diameter C-fibre neurons as determined by NF-200 immunostaining.

Finally, in order to further assess consequences of perineural HIV-1 gp120, we sought to assess whether a regenerative process was present in nerves one week or two weeks following treatment. Consistent with the findings of previous studies (Cafferty et al., 2004), we observe qualitatively three different levels of GAP43-IR. Under normal circumstances (i.e., in the absence of injury), a large percentage of cells express low levels of GAP43-IR (Van der Zee et al., 1989; Schreyer and Skene, 1991) with a small percentage expressing elevated GAP43-IR levels. Elevated expression levels are only seen significantly after a peripheral nerve injury in cells associated with regenerating axons. Therefore, as a positive control we assessed expression levels of GAP-43-IR in the ipsilateral DRG 7 days following partial sciatic nerve injury of the sciatic nerve (Seltzer et al., 1990). We used the results to set an automated detection level for positive neurons following injury, and counted as positive only those neuronal profiles which expressed similarly high levels of GAP-43 as an index of their enhanced regenerative potential. At 7 and 14 days following perineural HIV-1 gp120 there was no significant increase in the percentage of GAP-43-IR neurons in the DRG ipsilateral to HIV-1 gp120 treatment as compared to contralateral DRG or DRG ipsilateral to sham treatment (Table 1, Fig. 7A, d). This suggests that at times of peak behavioural sensitivity, nerves treated with HIV-1 gp120 are not associated with a significant axonal regenerative process. Furthermore, throughout all immunohistochemical analysis of the DRG, there was no significant difference in the total number of cells present in six sections of DRG for each of six animals in HIV-1 gp120 treatment group (average of 4970.6 ± 310 cells) versus sham (average of 4986.8 ± 140 cells) (see Tables 1 and 3 for counts), indicating a lack of overt neuronal loss in the DRG.

### 3.8 The effect of perineural HIV-1 gp120 on primary sensory neuronal phenotype

To determine whether perineural HIV-1 gp120 is associated with phenotypic changes in the DRG common to other models of nerve-injury induced pain, we have examined the expression of the TRPV1 receptor (Hudson et al., 2001) and the neuropeptides; CGRP, galanin and NPY (Villar et al., 1991; Hokfelt et al., 1994) at the time of peak behavioural change. By day 14, there was no overall change in the expression of TRPV1 in neurons in the DRG ipsilateral to perineural HIV-1 gp120 versus sham treatment or DRG contralateral to perineural HIV-1 gp120 (Table 3, Fig. 7B, a). Moreover, there was no significant difference between treatment groups in the percentage of small diameter, NF200 negative cells that were TRPV1-IR (gp120 = 52.5 ± 2.2% versus RSA = 57.8 ± 3.8%), indicating that the lack of overall TRPV1-IR change was not obfuscated by a change in the phenotype of TRPV1-IR cells. Likewise, there was no significant difference in the numbers of CGRP-IR neurons between the DRG ipsilateral and contralateral to perineural HIV-1 gp120 or as compared to sham treatment (Table 3, Fig. 7B, b). In contrast, we observed significant galanin-IR in 13.9% neurons ipsilateral to perineural HIV-1 gp120 treatment as compared to 3.2% neurons ipsilateral to RSA (sham) treatment and no significant IR contralateral to perineural HIV-1 gp120 (Table 3, Fig. 7B, c). Of the galanin-IR cells 87% (513 of 590) did not co-localise with NF200 suggesting the major population of galanin expressing cells are small diameter C-fibre neurons. There was also significant NPY-IR in approximately 12.2% of DRG neurons as opposed to 1.6% ipsilateral to RSA (sham) treatment and no detectable immunoreactivity in contralateral DRG (Table 2). All NPY-IR cells co-localised with NF-200-IR indicating that they were cells with myelinated fibres (Fig. 7B, d).

### 3.9 Alterations in non-neuronal cells in the DRG following perineural HIV-1 gp120

To determine whether morphological changes occur in satellite cells surrounding sensory neuronal cell bodies in the DRG, we assessed the expression of GFAP, the intermediate filament known to increase in expression following peripheral nerve injury (Woodham et al., 1989; Peters et al., 2005). Fourteen days following perineural HIV-1 gp120, there was a significant increase in proportion of neurons associated with GFAP-IR satellite cells in the DRG ipsilateral to perineural HIV-1 gp120 (11.6 ± 4.3%) as compared to DRG ipsilateral to RSA (sham) treatment (3.6 ± 0.6%) and contralateral DRG (1.6 ± 1.6%) (Fig. 7C, a).

Activated macrophages infiltrate injured peripheral nerve tissue and therefore can be considered as universal markers of PNS injury and are likely to be found following most PNS lesion (Myers et al., 1996; Caterina et al., 2000; Abbadie et al., 2003). Again using an antibody to CD68 (ED-1), we assessed the presence of activated macrophages in the DRG of perineural HIV-1 gp120 treated animals. At 14 days following treatment, there was a modest but significant increase in the average number of CD-68-IR macrophages present per section of the DRG ipsilateral to perineural HIV-1 gp120 (88.4 ± 7.4) as compared to contralateral DRG (36.4 ± 8.6) and DRG from sham treated animals (39.3 ± 4.7) (Fig. 7C, b).

### 3.10 Spinal gliosis following perineural HIV-1 gp120

**Fig. 8:**
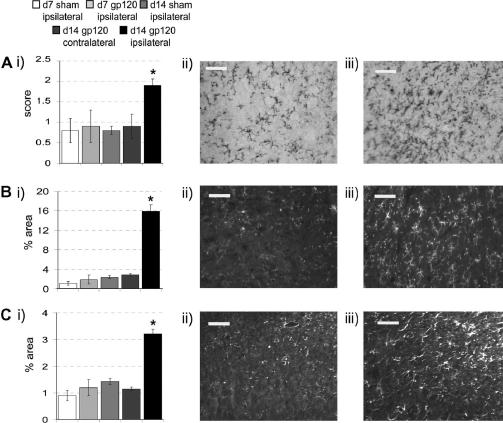
Immunohistochemical analysis of microglia and astrocytes in the dorsal horn of the L5 spinal cord 14 days following perineural HIV-1 gp120. (i) There is a statistically significant increase in the (A) morphological rating of microglia, (B) density of OX-42-IR and (C) density of GFAP-IR in L5 spinal cord dorsal horn ipsilateral versus contralateral to perineural HIV-1 gp120 at day 14 (*n* = 6) and compared to the dorsal horn ipsilateral to perineural RSA (sham) at day 14 (*n* = 6) and ipsilateral to perineural HIV-1 gp120 or RSA at day 7 (*n* = 6). Statistical significance of differences between groups (*P* < 0.05) was determined by an ANOVA with Dunn’s all pairwise multiple comparisons where ∗ represents significant difference from all other groups. Each value is means ± SEM. (ii and iii) Examples of the expression of (A) OX-42-IR with biotin labelling for qualitative analysis in the L5 spinal cord dorsal horn, (B) OX-42-IR with fluorescence labelling for quantitative analysis in the dorsal horn of the L5 spinal cord, and (C) GFAP-IR with fluorescence labelling in the L5 spinal cord dorsal horn at day 14 (ii) ipsilateral to perineural RSA and (iii) ipsilateral to perineural HIV-1 gp120. Scale bar = 50 μm.

In order to gain a better understanding of the central consequences of peripherally applied HIV-1 gp120, as well as to reproduce changes previously reported in a similar model (Herzberg and Sagen, 2001), we assessed the presence of morphological and population changes in microglia and astrocytes in the dorsal horn of the spinal cord, by measurement of OX-42-IR and GFAP-IR, respectively. We examined two time points; day 7 following surgery, a time when behavioural changes were minimal and day 14 following surgery, by which time behavioural changes had peaked. We employed a rating scale of 0–3 to assess the activation state of microglia (adapted from (Colburn et al., 1997)), where 0 indicates the resting state and 3 represents a highly activated state. At day 14, microglia in the dorsal horn ipsilateral to perineural HIV-1 gp120 scored 2.1 ± 0.2 versus 0.9 ± 0.3 contralaterally, indicating that there was a significant qualitative, morphological change. Such a change was not apparent at day 7 following perineural gp120 or in sham treated animals (Fig. 8A and B). We also assessed the density of microglia present by measuring the percentage of a defined area expressing OX-42-IR (Blackbeard et al., 2005). At day 7 there was a small but significant increase in the density of microglia in the ipsilateral dorsal horn as compared to sham (Fig. 8A). Whereas, by day 14, there was a 5-fold increase in the density of microglia in the dorsal horn ipsilateral to perineural HIV-1 gp120 as compared to sham values (Fig. 8A and C). There was no such change contralateral to treatment. By day 14, HIV-1 gp120-treated rats also exhibited a 5-fold increase in the density of astrocytes present in the dorsal horn ipsilateral to treatment versus the contralateral dorsal horn or versus sham values (Fig. 8A and D). No such change was present by day 7 following treatment.

## 4 Discussion

Over one-third of people living with HIV suffer painful peripheral neuropathies (Verma et al., 2005; Marshall et al., 2005), the mechanisms of which are poorly understood. The HIV-1 coat protein, gp120, has been linked to genesis of neuropathic pain (Oh et al., 2001; Keswani et al., 2003; Melli et al., 2006). To ascertain the biological capacity of gp120 to affect the nerve *in vivo* and so investigate the possible mechanisms involved in the development of neuropathic changes, we have pharmacologically and mechanistically characterised a rodent model of HIV-1 gp120 induced neuropathic pain.

HIV-1 gp120 induces mechanical hypersensitivity with a time course that differs in onset and duration to other nerve-lesion pain models (Devor and Seltzer, 1999; Decosterd and Woolf, 2000). In further contrast to many nerve-lesion models, but in line with clinical data from patients with HIV-related painful neuropathy (Martin et al., 2003), no hypersensitivity to heat or cold stimuli developed. The hypersensitivity correlates with decreased spontaneous exploratory behaviour and thigmotaxis in the open field, behaviour thought to correlate to anxiety in rodents (Cryan and Holmes, 2005). This suggests the presence of alterations in emotionally driven behaviour and may represent a pain-related co-morbidity that can be utilised in the future.

To assess the suitability of this as a model in which to study neuropathic pain mechanisms, we investigated the effects of known analgesic compounds. Amitriptyline has known analgesic efficacy in neuropathic pain in experimental models (Abdi et al., 1998; De Vry et al., 2004) and in the clinic (Hempenstall et al., 2005; Finnerup et al., 2005). However, clinical trial data suggest that amitriptyline is not effective for the treatment of HIV related neuropathic pain (Kieburtz et al., 1998; Shlay et al., 1998), and amitriptyline had no effect on mechanical hypersensitivity in our model. In contrast, gabapentin and morphine were both effective in our model. Gabapentin is analgesic in rodent (Abdi et al., 1998; De Vry et al., 2004; Donovan-Rodriguez et al., 2005) and human neuropathic pain conditions (Hempenstall et al., 2005; Finnerup et al., 2005), including HIV-neuropathy (Hahn et al., 2004). Morphine attenuates hypersensitivity in rodent neuropathy models (Lee et al., 1994; Backonja et al., 1995) and evidence is accumulating to suggest that opioids relieve symptoms of human neuropathic pain of peripheral origin (Hempenstall et al., 2005; Finnerup et al., 2005). The effects of these analgesics in our model correlate well with their clinical efficacy further supporting the use of this model for the assessment of novel therapeutic agents. For example, evidence indicates that cannabinoids may exert beneficial effects including analgesia in human neuropathic pain (Rice, 2005; Finnerup et al., 2005). Here we have shown that in line with previous rodent neuropathic pain studies (Bridges et al., 2001; Farquhar-Smith et al., 2002; Wallace et al., 2003), the CB_1_/CB_2_ receptor agonist, WIN 55,212-2, effectively attenuated HIV-1 gp120 induced mechanical hypersensitivity further supporting the potential therapeutic benefit of cannabinoids.

To understand mechanisms of HIV-1 gp120 related neuropathic behaviour, we sought to establish whether axonal damage is a consequence of gp120 application. LM and EM investigations revealed relatively few demyelinating and degenerating large diameter axons and little evidence for damage to unmyelinated axons at sites distal to or at the application site. Additionally, the lack of increased expression of the axonal regeneration marker GAP-43 (Schreyer and Skene, 1991), as well as a lack of macrophages in the distal nerve, indicate that widespread axonal damage and regeneration, especially of larger diameter fibres, is not a significant consequence of this model. The absence of axonal degeneration in the peripheral nerve does not rule out the possibility of a degenerative process in the sensory neuron peripheral terminal arbors, which has been shown to be a feature of alternative models of neuropathy (Siau et al., 2006). Quantification of intraepidermal nerve fibre density in the plantar hind paw revealed gp120 treated nerves to have reduced numbers of C-fibre peripheral terminals at times of peak behavioural sensitivity. IENF density is commonly used for diagnosis of sensory neuropathy (McCarthy et al., 1995; Kennedy et al., 1996; Lin et al., 2001) and is inversely correlated with the presence of neuropathic pain in HIV patients (Polydefkis et al., 2002). Furthermore, the cell stress marker, ATF3 (Tsujino et al., 2000), was significantly expressed at this time particularly in small diameter sensory neurons. These results reflect the clinical scenario in which HIV-DSP is thought to be a predominantly small fibre neuropathy affecting the distal nerve (Martin et al., 2003; Verma et al., 2005) and are in line with recent *in vitro* evidence (Melli et al., 2006) supporting neurotoxicity of gp120 on axons. This may represent a mechanism by which HIV infection results in axonopathy in humans and further supports the use of this model in the investigation of such mechanisms.

The presence of macrophage infiltrates within the peripheral nerve and DRG of HIV/AIDS patients, with concomitant presence of pro-inflammatory cytokines (de la Monte et al., 1988; Yoshioka et al., 1994; Nagano et al., 1996; Pardo et al., 2001), links HIV-1 infection with an inflammatory response within the nerve and DRG. Correlating with the clinical scenario, significant numbers of macrophages are present in the gp120 treated nerve in a time course consistent with hypersensitivity. Macrophage derived inflammatory mediators likely influence nerve function and associated sensory abnormalities observed in this model and may therefore emerge as useful therapeutic targets.

Primary sensory neuron gene expression changes are known consequences of nerve injury and may contribute to peripheral and central plasticity (Costigan et al., 2002). At the time of peak behavioural change we found no change in DRG expression of CGRP or TRPV1. Dysregulation of both proteins following peripheral nerve injury (Villar et al., 1991; Hudson et al., 2001) has been suggested to correlate with the development of thermal hyperalgesia (Caterina et al., 2000; Mogil et al., 2005), a phenomenon that is neither a feature of our model, nor of clinical data (Martin et al., 2003). However, the expression of galanin, NPY; the satellite cell marker, GFAP and the macrophage marker, CD68, did increase in the DRG, all of which are features of models of nerve-lesion associated pain (Ma and Bisby, 1998; Wynick et al., 2001). Although of a smaller magnitude than seen in nerve lesion models, these changes suggest that HIV-1 gp120 in the nerve can produce changes usually associated with actual nerve trauma and may further reflect gp120 induced axonal injury.

A number of studies have linked gp120 to induction of apoptosis in neurons (Lipton, 1991; Keswani et al., 2003; Melli et al., 2006). The pro-apoptotic proteins c-Jun (Ham et al., 2000) or caspase-3 (Beattie et al., 2002) elicit apoptosis in various cells, and can increase following nerve injury (De Felipe and Hunt, 1994; Kenney and Kocsis, 1998; Jiang et al., 2005) or exposure to gp120 *in vitro* (Singh et al., 2005). We found that caspase-3, but not p-c-Jun, increased in the DRG suggesting that specific apoptotic pathways are induced by HIV-1 gp120. The expression of caspase-3, particularly in small diameter neurons, implies that these cells may undergo apoptosis with the resultant loss of small calibre axons at later time points. As mentioned above, HIV-DSP presents as a small fibre neuropathy and such mechanisms may well contribute and therefore merit further investigation. Furthermore, these data correlate well with *in vitro* studies which link gp120 induced apoptosis to caspase-3 dependent mechanisms (Keswani et al., 2003; Melli et al., 2006) and indicate that this model lends itself to the investigation of the biological effect of gp120 on peripheral axons. Therefore, although not a direct model of HIV-associated neuropathy in man, the current model will likely prove useful in future studies to unravel mechanisms of gp120 related neuropathic events.

Finally, we have demonstrated spinal gliosis in a time course consistent with pain-like behaviour. Spinal astrocytes and microglia can produce and/or respond to a number of pronociceptive substances (Tsuda et al., 2005) and their activation has been demonstrated in various models of neuropathic pain (Colburn et al., 1997; Milligan et al., 2003; Tanga et al., 2004). This suggests a mechanism by which HIV-1 gp120 can induce long-lasting central neuropathology in the absence of direct CNS infection and may point towards potential therapeutic agents (Tsuda et al., 2005).

These findings lead to important questions as to the mechanisms by which HIV-1 gp120 induces neuropathic changes and pain. HIV-1 gp120 binds to the chemokine receptors CXCR4 and CCR5, the natural ligands of which (SDF-1a and RANTES) can induce behavioural correlates of pain in rats (Oh et al., 2001). Both receptors are expressed on rat DRG neurons (Oh et al., 2001), axons (Melli et al., 2006) and by macrophages (Lee et al., 2003). We have demonstrated the presence of macrophages in the HIV-1 gp120 treated nerve as well as correlates of axonal injury at peripheral terminals and in the DRG implying that these receptors are feasible sites of action in this model. However, this mechanism of HIV-1 gp120 induced neuropathic changes has yet to be proven and this study suggests that this model would lend itself to such investigation.

In conclusion, we have characterised a neuropathic pain model that mimics many features of HIV-DSP. The associated characteristics, especially the robust but specific behavioural changes, highlight the utility of this model for many further mechanistic studies and potentially the discovery of new therapeutic targets for the treatment of neuropathic pain.

## Figures and Tables

**Table 1:** ATF3 and GAP-43 immunoreactivity in DRG cells following perineural HIV-1 gp120 treatment

	HIV-1 gp120		RSA sham
	Ipsilateral	Contralateral	Ipsilateral
ATF3 day 7	8.6 ± 1.9%^∗^ (440 of 5126)	0.7 ± 0.6% (36 of 5231)	3.6 ± 1.9%^‡^ (183 of 5110)
ATF3 day14	26.9 ± 2.9%^∗^ (1483 of 5502)	0.8 ± 0.6% (4 of 4960)	4.6 ± 2.4%^‡^ (240 of 5236)
GAP43 day 7	2.6 ± 0.4% (91 of 3624)	2.7 ± 0.9% (111 of 4120)	2.6 ± 0.6% (113 of 4351)
GAP43 day 14	3.3 ± 1.8% (144 of 4977)	3.4 ± 0.7% (157 of 4624)	1.8 ± 0.6% (82 of 4922)

The expression of ATF3 and GAP-43 immunoreactivity as a percentage of total DRG cells ipsilateral and contralateral to HIV-1 gp120 treatment of the sciatic nerve and ipsilateral to perineural RSA (sham) on post treatment day 14 (*n* = 6 in each case). Data are presented as means ± SEM. Total cell counts are in brackets. There was a significant increase in the expression of ATF3 at day 7 and day 14 post treatment in the DRG ipsilateral to perineural HIV-1 gp120 as compared to contralateral and sham DRG (^∗^*P* < 0.05) as determined by a Kruskal–Wallis one-way ANOVA on Ranks with Dunn’s post hoc analysis. There was a small but significant increase in ipsilateral sham DRG as compared to contralateral DRG (^‡^*P* < 0.05).

**Table 2:** P-c-Jun and caspase-3 immunoreactivity in DRG cells following perineural HIV-1 gp120 treatment

	HIV-1 gp120		RSA sham
	Ipsilateral	Contralateral	Ipsilateral
p-c-Jun day 14	13.8 ± 0.7% (257 of 1965)	12.3 ± 2.6% (244 of 1975)	11.5 ± 0.8% (223 of 1998)
Caspase-3 day 14	25.3 ± 1.8%^∗^ (854 of 3420)	0% (0 of 3498)	0% (0 of 3268)

The expression of p-c-Jun and caspase-3 immunoreactivity as a percentage of total DRG cells ipsilateral to HIV-1 gp120 or RSA treatment of the sciatic nerve post treatment day 14 (*n* = 4 in each case). Data are presented as means ± SEM. Total cell counts are in brackets. There was a significant increase in the expression of caspase-3 in the DRG ipsilateral to perineural HIV-1 gp120 as compared to sham DRG (^∗^*P* < 0.05) as determined by a Kruskal–Wallis one-way ANOVA on Ranks with Dunn’s post hoc analysis.

**Table 3:** Immunohistochemical assessment of neuronally expressed proteins in DRG cells 14 days following perineural HIV-1 gp120 treatment

	HIV-1 gp120		RSA sham
	Ipsilateral	Contralateral	Ipsilateral
TRPV1 day 14	35.6 ± 1.9% (2347 of 6562)	38.2 ± 2.6% (1962 of 5136)	32.8 ± 1.8% (1886 of 5624)
CGRP day 14	28.4 ± 2.5% (1306 of 4694)	32.3 ± 2.6% (1453 of 4498)	33.4 ± 1.6% (1633 of 4842)
Galanin day 14	13.9 ± 1.3%^∗^ (590 of 4374)	0% (0 of 4672)	3.2 ± 1.5%^‡^ (210 of 5308)
NPY day 14	12.2 ± 1.9%^∗^ (838 of 4368)	0% (0 of 4310)	1.6 ± 0.3%^‡^ (72 of 4502)

The expression of TRPV1, CGRP, galanin and NPY immunoreactivity as a percentage of total DRG cells ipsilateral and contralateral to HIV-1 gp120 treatment of the sciatic nerve and ipsilateral to perineural RSA (sham) on post treatment day 14 (*n* = 6 in each case). Data are presented as means ± SEM. Total counts are in brackets. There was no significant change in TRPV1-IR or CGRP-IR between groups. There was a significant increase in the expression of galanin and NPY in the DRG ipsilateral to perineural HIV-1 gp120 as compared to contralateral and sham DRG (^∗^*P* < 0.05) as determined by a Kruskal–Wallis one-way ANOVA on Ranks with Dunn’s post hoc analysis. A small but significant increase was also observed in ipsilateral sham DRG as compared to contralateral DRG (^‡^*P* < 0.05). There was no difference in the number of cells expressing either CGRP or TRPV1 between groups as determined by a Kruskal–Wallis one-way ANOVA on Ranks.
